# Epidemiology and Genotype Distribution of Hepatitis C Virus in Russia

**DOI:** 10.3390/pathogens11121482

**Published:** 2022-12-06

**Authors:** Nikolay Pimenov, Dmitry Kostyushev, Svetlana Komarova, Anastasia Fomicheva, Alexander Urtikov, Olga Belaia, Karina Umbetova, Olga Darvina, Natalia Tsapkova, Vladimir Chulanov

**Affiliations:** 1 National Medical Research Center of Phthisiopulmonology and Infectious Diseases, Moscow 127473, Russia; 2 Laboratory of Genetic Technologies, Martsinovsky Institute of Medical Parasitology, Tropical and Vector-Borne Diseases, I.M. Sechenov First Moscow State Medical University (Sechenov University), Moscow 119991, Russia; 3 Division of Biotechnology, Sirius University of Science and Technology, Sochi 354340, Russia; 4 Department of Infectious Diseases, I.M. Sechenov First Moscow State Medical University (Sechenov University), Moscow 119991, Russia; 5 F. Erisman Institute of Public Health, I.M. Sechenov First Moscow State Medical University (Sechenov University), Moscow 119991, Russia

**Keywords:** hepatitis, HCV, epidemiology, genotype, subgenotype, recombinant, RF1_2k/1b, direct-acting antivirals

## Abstract

The hepatitis C virus (HCV) causes both acute and chronic infection of the liver that can lead to liver cirrhosis, cancer, and liver failure. HCV is characterized by high genetic diversity and substantial variations in the prevalence of specific HCV genotypes throughout the world. Many effective regimens of direct-acting antivirals (DAAs), including pan-genotypic, can successfully treat HCV infection. Additionally, genotype-specific treatments for HCV are being actively employed in national plans for eliminating HCV infection around the world. The evaluation of HCV genotype prevalence in a given country is necessary for the successful implementation of the HCV elimination plans and for allocating financial resources to the DAAs which are the most effective against those specific HCV genotypes prevalent in a given country. Here, we analyzed HCV genotypes, subgenotypes, and recombinants in 10,107 serum samples collected in 2015–2017 from patients with chronic HCV infection living in all federal districts of Russia. This is the first and largest evaluation of HCV genotypes performed on samples from all territories of Russia, from its Central federal district to the Far East. Moreover, we have updated retrospective epidemiological analysis of chronic and acute HCV infection in Russia from 2001 to 2021. We demonstrate that the incidence of acute HCV (AHC) infection in Russia decreased from 16.7 cases per 100,000 people in 2001 to 0.6/100,000 in 2021. The number of cases of chronic HCV (CHC) infection also decreased from 29.5 to 16.4 per 100,000 people during this period. The HCV genotype analysis indicated that HCV genotype 1 dominates in Russia (53.6%), while genotypes 3 and 2 were detected in 35.4% and 7.8% of patients, respectively. These proportions are virtually identical in all regions of Russia except for the Far East, where HCV genotype 2 was detected in only 1% of the samples. HCV genotypes 1 and 2 are more widespread in women, and HCV genotype 3 in men. Genotype 3 was the most prevalent in 31–40-year-olds (44.9%), and genotype 1 was most prevalent in those over 70 years of age (72.2%). HCV genotype 2 was predominant among HCV-infected persons older than 40 years. Discriminating between HCV genotype 2 and recombinant RF1_2k/1b, which are frequently misclassified, is important for successful antiviral treatment. For the first time, we demonstrate, here, countrywide prevalence of HCV RF1_2k/1b in different regions of Russia. HCV RF1_2k/1b makes up 3.2% of HCV genotypes, reaching 30% among samples classified as genotype 2 by some commercial genotyping tests. The highest proportion of HCV RF1_2k/1b was detected in the North-West (60%), Southern (41.6%), and Central (31.6%) federal districts; its frequency in the Far Eastern and North Caucasus districts was ~14.3%. HCV RF1_2k/1b, and it was not detected in the Volga, Ural, or Siberian districts. To conclude, this is the first and most complete evaluation of HCV epidemiology and genotype/subgenotype distribution in Russia.

## 1. Introduction

Acute and chronic hepatitis C infections are caused by the hepatitis C virus (HCV), which infects the liver and induces liver inflammation. HCV is an enveloped, plus-strand RNA virus of a *Flaviviridae* family. HCV infection can lead to severe liver diseases, including liver cirrhosis (LC) and cancer (hepatocellular carcinoma, HCC). It is a global health problem and, along with hepatitis B virus (HBV) infection, is the leading cause of cirrhosis and liver cancer in the world [1]. In 2015, 71 million people worldwide were estimated to live with HCV infection, while the global prevalence of infection was 1% [1]. According to the World Health Organization’s (WHO) latest estimates, 58 million people have chronic hepatitis C (CHC), with ~1.5 million new infections occurring every year. Especially concerning is that an estimated 3.2 million adolescents and children have CHC. In 2019, approximately 290,000 people died due to consequences of HCV infection, mostly LC and HCC [2]. Numerous non-hepatic manifestations of HCV infection have also been reported. HCV infection is associated with various heart abnormalities (cardiomyopathies, myocarditis, arrhythmias, etc.) [3], abnormalities of the kidneys [4], pathogenesis of diabetes, atherosclerosis [5], and many types of cancers (pancreatic, blood cancers, etc.) [6,7,8,9].

To eliminate hepatitis as a threat to public health, the 69th World Health Assembly adopted the first global health sector strategy, aiming to reduce new HBV and HCV infections by 90% and deaths by 65% by 2030, compared to 2015 [10,11]. The WHO Regional Office for Europe approved the Action Plan for the health sector response to viral hepatitis. According to the goals adopted by the WHO Regional Office for Europe, by 2030, 50% of all people living with chronic viral hepatitis B, C, and D, and 75% of those with LC or HCC, should be diagnosed, 75% of HCV patients meeting the criteria for treatment should receive antiviral therapy, and at least 90% of HCV patients should be completely cured of the infection [12].

HCV displays a high genetic diversity, and is currently classified into eight genotypes (GTs), with varying geographic prevalence in different regions of the world [13]. Globally, genotypes 1, 3, and 4 are the most common, representing 44%, 25%, and 15% of all HCV cases, correspondingly. Genotype 1 is dominant (60%) in high- and middle-income countries, genotype 3 (36%) is most common in middle-income countries, and genotype 4 (45%) prevails in low-income countries [14].

For many years, various direct-acting antiviral drugs (DAA) have been successfully used to treat HCV infection. Pangenotypic and genotype-specific treatment regimens for HCV infection have been developed [15]. Although HCV can currently be cured with new DAAs, many countries are facing challenges associated with the late diagnosis of HCV infection and poor access to treatments. As a result, HCV infection is often diagnosed at late stages of the disease, when liver damage is severe [16].

HCV infection has been registered in all regions of the world; however, about 80% of infected persons live in just 30 countries [14]. A large number of HCV-infected persons live in Russia [17]. The current population of Russia is over 146 million people (ninth most populous country in the world and most populous in Europe). The average age in Russia is 39 years. The average population density is 8.6 people/km² (highest in Moscow with 4950 people/km²; lowest in the Chukchi autonomous district with 0.1 people/km²). Most (74.6%) of the population is urban [18]. Russia’s eight federal districts (Central, Northwestern, Volga, Ural, North Caucasian, Southern, Siberian, and Far Eastern) are highly diverse ethnically, geographically, and culturally, with different migration patterns and travel history that may all affect the prevalence of HCV genotypes [18]. The high prevalence of HCV infection, along with the unique geographical and demographic features of Russia, creates many challenges for analyzing the distribution of HCV infection in the country and meeting the goal for eliminating HCV infection as a serious public health threat by 2030. Despite the reduction in HCV infection in Russia, chronic infection remains a serious healthcare problem. To provide medical assistance to patients with CHC in Russia and develop a strategy for treating such patients with modern antivirals, it is necessary to understand the epidemiology of HCV infection in this country.

Until now, HCV genotype distribution in all Russian regions has never been studied. Only several reports on HCV genotypes in small sample groups and with specific groups of patients have been published previously [19,20,21,22]. These studies demonstrated the major prevalence of the HCV 1b genotype (68.9% to 76% according to different reports) and, additionally, circulation of the HCV genotypes 1a, 2, and 3.

The aim of our study was to evaluate the current epidemiology of HCV infection in Russia and determine the distribution of HCV genotypes and clinically relevant HCV subgenotypes (1a, 1b), as well as recombinant RF1_2k/1b. Understanding HCV genotype distribution is extremely important for the rational use of DAAs and strategic planning of medical healthcare to ensure successful implementation of the HCV elimination program in a given country. Along with genotyping, we analyzed the distribution of HCV recombinant RF1_2k/1b, which is frequently misclassified as genotype 2 by some commercially available test kits, affecting the choice of DAAs and treatment outcomes. RF1_2k/1b is the most actively circulating in the world; however, other recombinants (2/5, 2b/1b, 2b/1a, 2i/6p) were described as well [23]. Highly effective sofosbuvir-based pan-genotypic treatment options have been proven to achieve high SVR in RF1_2k/1b. However, due to a low availability of pangenotypic treatments and implementation of cost-effective treatment strategies, differentiating RF1_2k/1b is important [24]. In particular, the sustained virologic response for recommended treatment regimens is substantially higher for HCV genotype 2 patients than for patients with the HCV RF1_2k/1b strain [25]. There are case reports that detail the cure rate of patients with RF_2k/1b and their treatment with the limited spectrum of DAAs. According to the available data on sustained virologic response (SVR) rates, patients with RF_2k/1b could be more similar to patients with GT1 than to patients with GT2 [24].

The RF1_2k/1b recombinant or «chimera» was first discovered in St. Peterburg by O. Kalinina et al. [26]. Although recombination in HCV genomes is extremely rare and plays only a minor role in HCV evolution, this particular recombinant has spread widely and is the most frequent HCV recombinant, although generally not exceeding 3% of other genotypes. RF1_2k/1b likely represents an HCV recombination event that occurred in the Soviet Union between 1923 and 1956 [27] and then spread across the world: the prevalence of RF1_2k/1b is 1.2% in Germany, 2.6% in Cyprus, 3% in Netherlands, 0.5% in Estonia [28], and 1% in Uzbekistan [29]. It has also been found in many other countries, including Moldova [30], Italy [31], Greece [32], and Austria [33]. The highest reported incidence of RF1_2k/1b was recently identified in Georgia, reaching ~20% [34]. Previous assessments with a limited sample size (285 samples) reported 2% prevalence of RF1_2k/1b in Russia [35]. In general, little is known about the prevalence of RF1_2k/1b in the world, and in Russia in particular. This is the first study providing a country-wide estimation of RF1_2k/1b recombinant distribution in Russia.

## 2. Materials and Methods

### 2.1. Retrospective Epidemiological Analysis

To estimate the incidence of AHC and CHC in Russia, we analyzed the official statistical data from 2001 to 2021 [36]. The analysis included the incidence of AHC and CHC in different age and sex groups.

### 2.2. Collection and Storage of Serum Samples

10,107 HCV positive serum samples were collected during routine epidemiological monitoring from 2015 to 2017. HCV isolates were obtained from patients from all 8 federal districts (35 regions in total) of Russia, and stored at −80 °C before use. All patients gave voluntary informed consent before participating in the study.

### 2.3. Isolation of HCV RNA

RNA was isolated from patient samples using commercial kits “RIBOSORB,” “MAGNO-sorb,” and “RIBOSOL E” (all from AmpliSens Biotechnologies, Moscow, Russia) according to manufacturer’s instructions. Reverse transcription was performed using “REVERTA-L” variant 100 kit (AmpliSens Biotechnologies, Moscow, Russia), designed to obtain cDNA on an RNA matrix according to the manufacturer’s instructions.

### 2.4. PCR Analysis and Genotyping of HCV

HCV cDNA was amplified by PCR using proprietary primers and reagents for amplification. HCV genotype was determined using the commercially available test kit “AmpliSens HCV-Genotype-FL” (AmpliSens Biotechnologies, Moscow, Russia). In brief, genotyping procedure is based on simultaneous identification of genotypes 1a, 1b, 2, 3, 4, 5, and 6 with specific sets of primers in several tubes using two fluorescent probes (FAM and JOE) together with an internal control using real-time PCR. All samples classified as genotype 2 were subsequently sequenced for confirmation purposes. “Rotor Gene Q” (Qiagen, Hilden, Germany) machine was used for real-time PCR analysis.

### 2.5. Sanger Sequencing

The RF_2k/1b HCV recombinant was determined by sequencing of core and NS5B regions of viral isolates classified as genotype 2 by commercially-available genotyping test. Sequence results were analyzed using Geneious software version 7.1.7 (Biomatters Limited, Auckland, New Zealand) and NCBI information resources (viral genotyping tool and BLAST).

### 2.6. Epidemiological Characterization of Samples from HCV-Infected Individuals

The study group included 5780 (57.2%) men and 4327 (42.8%) women aged 0 to 83 years (median age 41 years). All patients were divided into 8 age groups: under 15 years old, 16–20 years old, 21–30 years old, 31–40 years old, 41–50 years old, 51–60 years old, 61–70 years old, and over 70 years old.

### 2.7. Statistical Analysis

Data analysis was performed in SPSS software (SPSS 21.0.0.0). Chi-square tests were used to determine statistically significant differences between variables. *p* values < 0.05 were considered statistically significant.

## 3. Results

### 3.1. Epidemiology of HCV Infection in Russia

**Figure 1:**
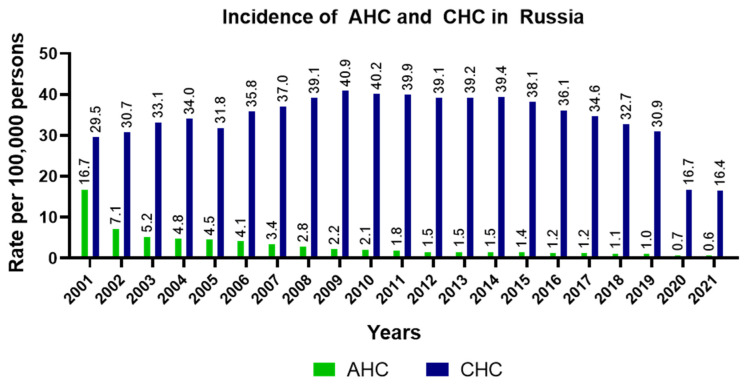
Incidence of acute and chronic hepatitis C in Russia in 2001–2021. The incidence rates of acute hepatitis C (AHC, green bars) and chronic hepatitis C (CHC, blue bars) per 100,000 persons.

Since the early 2000s, AHC incidence in Russia has shown a pronounced downward trend. In 2001, AHC incidence in Russia was 16.7 per 100,000 persons; by 2019, that rate dropped to 1/100,000 (Figure 1). On the other hand, the trends seen in the dynamics of CHC cases during that period of time are multidirectional. From 2001 to 2009, the CHC incidence rate increased, stabilized from 2010 to 2014, and decreased after 2015. In 2015, 55,596 cases of CHC were detected in Russia (38.1/100,000 persons), decreasing to 45,376 cases in 2019 (30.9/100,000 people). The sharp reduction in the incidence of CHC in 2020 and 2021 was most likely the result of low diagnosis and detection rates during the COVID-19 pandemic.

The incidence of hepatitis varied among Russia’s federal districts. In 2019, the highest number of CHC cases was registered in the Northwestern federal district (48.1/100,000 persons), and the lowest was registered in the North Caucasian district (12.9/100,000). The highest CHC incidence rates were registered among persons aged 30–39 years (94.4/100,000 in 2015) (results are not shown).

### 3.2. HCV Genotype and Subgenotype Distribution

**Figure 2:**
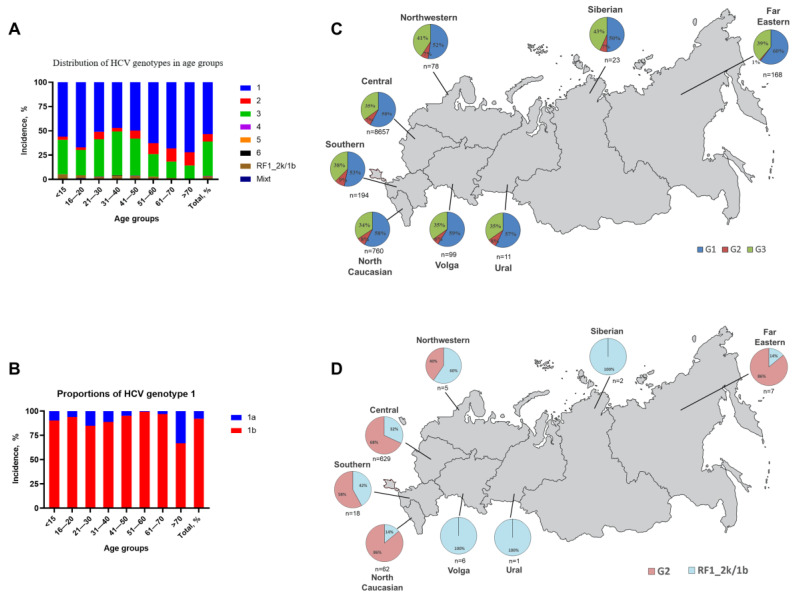
Molecular epidemiology of HCV in Russia. (**A**) Prevalence of HCV genotypes, recombinant, mixed genotypes, and (**B**) subgenotypes in different age groups. (**C**) Geographic distribution of HCV genotypes and (**D**) RF1_2k/1b recombinant.

This study showed that genotype 1 is predominant in Russia (53.6%) (Table 1, Figure 2A). Subtypes 1a and 1b were detected in 7.8% and 92.2% of genotype 1-positive samples, respectively (Figure 2B). Genotype 3 was detected in 35.4% of infected patients, and genotype 2, in 7.6%. Genotypes 4 and 6 were extremely rare (0.1–0.2%). Genotype 5 was not detected among the studied samples. The distribution of genotypes 1, 2, and 3 did not vary significantly between federal districts (*p* > 0.05), and the proportions of HCV genotypes remained virtually the same across Russia except for the Far East district, where genotype 2 was the lowest (1%) (Figure 2C). A mixture of two HCV genotypes was detected in 12 patients (0.1%) (Table 1).

**Figure 3:**
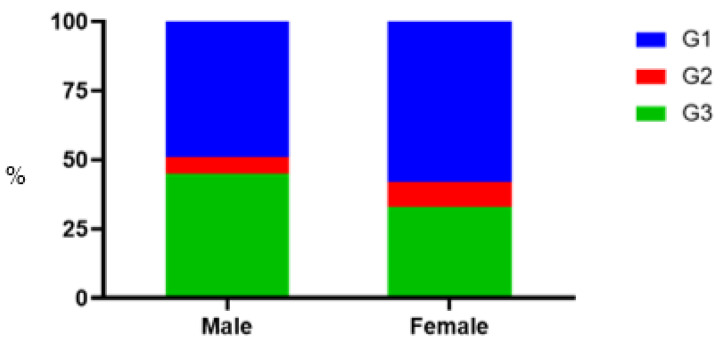
Differences in HCV genotype distribution between males and females.

The prevalence of HCV genotypes 1, 2, and 3 differed among gender and age groups (*p* < 0.05; Figure 2A,B and Figure 3, Table S1). The ratio of HCV genotypes 1, 2, and 3 was 49%, 6%, and 45% in men and 58%, 9%, and 33% in women, respectively (Figure 3, Table S1).

In all age groups, the proportion of genotype 1 was the largest. Among 31–40-year-olds, the proportion of genotype 1 was 47.1%, and of genotype 3, 44.9%. Among 41–50-year-olds, the frequency of genotype 3 was 38.1%, decreasing in older groups. On the contrary, the proportion of genotype 1 among 41–50-year-olds was 49.8% and increased with increasing age, reaching 72.2% among people over 70 years. The proportion of genotype 2 was lowest among people aged 16–20 years (2.7%) and increased in each subsequent age group, reaching its maximum value in 61–70-year-olds (13.5%).

### 3.3. Distribution of RF1_2k/1b Recombinant

RF1_2k/1b, the most prevalent HCV recombinant, was found in 312 infected patients, which amounted to 3.2% of the total genotypes and 30% of isolates classified as genotype 2 by commercial genotype testing (putative genotype 2) (Table 1, Figure 2A). The proportion of RF1_2k/1b recombinant among all age groups ranged from 0.9% to 5.4% (Table 1). Substantial differences were observed in its geographical distribution (*p* < 0.05) (Figure 2D), with the highest incidence in the North-Western (60% of putative genotype 2), Southern (41.6%), and Central (31.6%) federal districts (Figure 2D). In the Far Eastern and North Caucasus districts, the frequency of detection of the recombinant was 14.3% and 14.4% of HCV genotype 2, respectively. Although the recombinant was not detected in the Volga, Ural, and Siberian districts, this may be due to the small number of samples collected from these regions and the few cases of genotype 2 which were identified (*n* = 6, *n* = 1, and *n* = 2 in Volga, Ural, and Siberian districts, respectively).

The highest prevalence of the recombinant was identified among persons under 15 years of age (5.4%); however, it was generally similar in all age groups, descending from 3.6% in 41–50-year-olds to 0.9% in >70-year-olds (Table 1). The frequency of detection of the RF1_2K/1B recombinant differed significantly (*p* < 0.05) among men (3.9%) and women (2.4%).

## 4. Discussion

The high incidence of AHC in Russia in the early 2000s was related to the large number of intravenous drug users, most of which were young men [17,37]. Moreover, with no standardized methods for discriminating between acute and chronic infection, many cases of CHC were falsely diagnosed as AHC. In the subsequent years, as the prevalence of intravenous drug abuse in Russia declined, AHC incidence also dropped. In contrast, CHC incidence increased until 2009, then stabilized and started to decline only after 2015. Until 2020, over 40 thousand CHC cases were recorded annually in the country. In 2020–2021, when the COVID-19 pandemic began and quarantine measures were introduced, the number of new CHC cases decreased (~24,000 cases per year). In total, for 2001–2021, more than one million cases of hepatitis C were identified in Russia. The large number of new CHC cases has led to a high prevalence of CHC in the country [17].

HCV phylogenetic analyses identified similarities between Russian HCV isolates and those from Europe and Asia. RF1_2k/1b was found to be related to European strains [38]. The relative prevalence of HCV genotypes likely reflects the European influence and major socio-political, historical events associated with massive migration of the population [21,39,40].

According to WHO Guidelines, pangenotypic treatment regimens are prioritized for people with CHC. Genotype-specific treatments are recommended in countries where certain viral genotypes are more prevalent [25]. Several recent reviews extensively described available treatment options and the efficacy of genotype-specific and pangenotypic treatments [41,42,43]. Marcellusi et al. [44] recently estimated the cost consequence of investing into anti-HCV treatment access policies in Italy. It was demonstrated that the cost savings at a 20-year time horizon were ~€50–€55 million for 1000 treated patients. As such, along with substantial health benefits, the widespread use of anti-HCV therapies results in remarkable cost savings. According to these estimations, the time required for the cumulative costs saved to recover the initial investment of the Health System was estimated to be ~ 6.2–6.6 years. Therefore, there is an urgent need for and clear economic and health care benefits to implementing the national elimination program. 

The Russian national CHC guidelines include both pangenotypic regimens (velpatasvir + sofosbuvir; glecaprevir + pibrentasvir; daclatasvir + sofosbuvir) and various regimens for treating patients with subtypes 1a and 1b, and genotypes 3 and 4 [45]. The use of genotype-specific treatments in Russia is justified because the HCV genotype 1b strain is the most prevalent.

According to our study, HCV genotype 1 dominates in Russia (53.6%). Genotypes 3 and 2 were detected in 35.4% and 7.8% of patients, respectively (Table 1). Over 92.1% of all HCV genotype 1 samples were of subtype 1b (Table 1). The distribution of HCV genotypes is very uniform across the country, with similar proportions of the three major genotypes across all districts (Figure 2C). This is a refined, and the most complete, evaluation of HCV genotype distribution performed across all regions of Russia, with the largest set of patients’ samples.

Genotypes 1 and 2 HCV were more often detected in women, and genotype 3 in men. Genotype 3 was found most frequently in the age group 31–40-year-olds (44.9%), and genotype 1 in those over 70 (72.2%). The proportion of genotype 2 was higher among those over 40. HCV RF1_2k/1b prevalence was 3.2%, which is higher than in previous estimates (1.5–2%) [26,29].

The Russian Ministry of Health has prepared a national plan for HCV elimination, a comprehensive program that covers all aspects, ranging from HCV prevention to the treatment and control of HCV infection, with the final goal being to substantially reduce the incidence and mortality of HCV infection by 2030. Thus, the data obtained during the study on the distribution of HCV genotypes in the country has become the basis for meeting the needs of genotype-specific therapeutic regimens.

To achieve the WHO’s goals of HCV elimination by 2030, further work is needed to expand testing and treatment. All patients with CHC should be given DAAs to significantly reduce CHC prevalence and mortality from liver cirrhosis and primary liver cancer. A comprehensive treatment program will reduce the socio-economic burden of hepatitis C and achieve the targets of the WHO strategy for eliminating HCV infection in Russia by 2030.

Although the incidence of AHC and CHC in Russia has been declining in recent years, a large number of patients with CHC require antiviral treatment in accordance with modern national guidance. In Russia, a comprehensive national hepatitis C plan has been prepared, which includes all aspects from prevention to the treatment and control of diseases to reduce incidence and mortality from HCV infection by 2030.

## 5. Conclusions

To conclude, HCV infection remains a global health problem Achieving the WHO Plans for eliminating HCV as a public health threat by 2030 requires extensive characterization of HCV prevalence and genotype characteristics (distribution of HCV genotypes, subgenotypes, and RF1_2k/1b recombinant). Here, we demonstrate a very uniform pattern of HCV genotype distribution across the country, with the highest prevalence of HCV genotype 1 (53.6%) followed by genotypes 3 and 2. HCV genotypes 4 and 6, and mixed genotypes, were extremely rare (~0.1–0.2%), while genotype 5 was absent. The prevalence of RF1_2k/1b recombinant was higher compared to previous estimations. Significant differences in HCV genotypes were found between gender and age groups. Overall, these data provide the basis for implementing national plans for eliminating HCV in Russia.

## Figures and Tables

**Table 1:** Prevalence of different HCV genotypes, sub-genotypes, and recombinant RF1_2k/1b in Russia.

Age Groups	Genotype (or Subtype) Total Value (Percentage %)								
	1		2	3	4	5	6	RF1_2k/1b	Mixed
	1a	1b							
<15	7 (5, 4)	66 (50, 8)	4 (3, 1)	46 (35, 4)	0 (0, 0)	0 (0, 0)	0 (0, 0)	7 (5, 4)	0 (0, 0)
16–20	3 (4, 1)	46 (63, 0)	2 (2, 7)	19 (26, 0)	0 (0, 0)	0 (0, 0)	0 (0, 0)	2 (2, 7)	1 (1, 4)
21–30	83(7, 7)	463 (43, 2)	82 (7, 7)	412 (38, 5)	2 (0, 2)	0 (0, 0)	0 (0, 0)	29 (2, 7)	0 (0, 0)
31–40	188 (5, 3)	1493 (41, 8)	129 (3, 6)	1604 (44, 9)	8 (0, 2)	0 (0, 0)	6 (0, 2)	134 (3, 8)	7 (0, 2)
41–50	54 (2, 4)	1078 (47, 4)	189 (8, 3)	866 (38, 1)	5 (0, 2)	0 (0, 0)	0 (0, 0)	81 (3, 6)	1 (0, 0)
51–60	9 (0, 5)	1107 (62, 2)	202 (11, 3)	409 (23, 0)	0 (0, 0)	0 (0, 0)	0 (0, 0)	51 (2, 9)	2 (0, 1)
61–70	19 (2, 2)	570 (66, 0)	116 (13, 4)	145 (16, 8)	1 (0, 1)	0 (0, 0)	0 (0, 0)	12 (1, 4)	1 (0, 1)
>70	55 (23, 9)	111 (48, 3)	31 (13, 5)	31 (13, 5)	0 (0, 0)	0 (0, 0)	0 (0, 0)	2 (0, 9)	0 (0, 0)
Total	418 (4, 2)	4934 (49, 4)	755 (7, 6)	3532 (35, 4)	16 (0, 2)	0 (0, 0)	6 (0, 1)	318 (3, 2)	12 (0, 1)

## Supplementary Materials

The following supporting information can be downloaded at: https://www.mdpi.com/article/10.3390/pathogens11121482/s1, Table S1: Prevalence of HCV genotypes in males and females.
