# Supplementary material for: Epidemiology and Genotype Distribution of Hepatitis C Virus in Russia
Source: Pathogens. 2022 Dec 6;11(12):1482. doi: 10.3390/pathogens11121482 (PMC9781887; doi:10.3390/pathogens11121482)
Supplement: Supplementary file 1 [file pathogens-11-01482-s001.zip › pathogens-2036479-supplementary.pdf]

Table S1. Prevalence of HCV genotypes in males and females.

Genotype	Sex total value (percentage %)	
	**Male**	**Female**
G1	2701 (49)	2394 (58)
G2	331 (6)	371 (9)
G3	2480 (45)	1362 (33)
